# No Causal Association Between Adiponectin and the Risk of Rheumatoid Arthritis: A Mendelian Randomization Study

**DOI:** 10.3389/fgene.2021.670282

**Published:** 2021-09-24

**Authors:** Hanzhu Chen, Shuai Mi, Jiahao Zhu, Weidong Jin, Yasong Li, Tianle Wang, Yingjun Li, Chunhong Fan

**Affiliations:** ^1^School of Public Health, Hangzhou Medical College, Hangzhou, China; ^2^Department of Clinical Laboratory, Zhejiang Provincial People’s Hospital, People’s Hospital of Hangzhou Medical College, Hangzhou, China; ^3^Department of Rheumatology and Immunology, Zhejiang Provincial People’s Hospital, People’s Hospital of Hangzhou Medical College, Hangzhou, China

**Keywords:** rheumatoid arthritis, adiponectin, causality, Mendelian randomization, bidirectional

## Abstract

**Background:** Accumulating evidence from observational studies suggested that circulating adiponectin levels are associated with the risk of rheumatoid arthritis (RA), but the causality remains unknown. We aimed to assess the causal relationship of adiponectin with RA risk.

**Methods:** Based on summary statistics from large-scale genome-wide association studies (GWAS), we quantified the genetic correlation between adiponectin and RA. Then bidirectional Mendelian randomization (MR) analysis was performed to assess the causal relationship. Twenty single-nucleotide polymorphisms (SNPs) associated with adiponectin were selected as instrumental variables from a recent GWAS (*n* = 67,739). We applied theses SNPs to a large-scale GWAS for RA (14,361 cases and 43,923 controls) with replication using RA data from the FinnGen consortium (6,236 cases and 147,221 controls) and the UK Biobank (5,201 cases and 457,732 controls). The inverse-variance weighted (IVW) and multiple pleiotropy-robust methods were used for two-sample MR analyses.

**Results:** Our analyses showed no significant genetic correlation between circulating adiponectin levels and RA [*rG* = 0.127, 95% confidence interval (CI): –0.012 to 0.266, *P* = 0.074]. In MR analyses, genetically predicted adiponectin levels were not significantly associated with the RA risk (odds ratio: 0.98, 95% CI: 0.88–1.09, *P* = 0.669). In the reverse direction analysis, there is little evidence supporting an association of genetic susceptibility to RA with adiponectin (β: 0.007, 95% CI: –0.003 to 0.018, *P* = 0.177). Replication analyses and sensitivity analyses using different models yielded consistent results.

**Conclusions:** Our findings provided no evidence to support the causal effect of adiponectin levels on RA risk and of RA on circulating adiponectin levels.

## Introduction

Rheumatoid arthritis (RA) is an autoimmune disease with unclear etiology, mainly involving synovial joints, leading to joint malformation and a reduced quality of life (Lee and Bae, 2018). RA can be distributed at any age, and the high incidence age is 40–60 years old. The etiology of RA remains vague, but both genetic and environmental triggers likely result in the onset and progression of the disease (Bae and Lee, 2019a).

The most abundant adipokines in plasma is adiponectin, an endogenous bioactive polypeptide or protein secreted only by adipose tissue (Li and Wu, 2012; Dan et al., 2020). It stimulates endothelial cells, monocytic cells, and synovial fibroblasts to product interleukin-6 (IL-6) and metalloproteinase (MMP) (Lee and Bae, 2018). Moreover, adiponectin is presumed to be closely associated with the adjustment of inflammatory responses (Otero et al., 2006). Lots of studies have been conducted to explore the relationship between adiponectin and RA risk, but the results are inconsistent. Some studies had showed that circulating adiponectin levels in RA patients were higher than those in healthy controls (Lee and Bae, 2018), while others found an opposite significant or either null association (El-Hini et al., 2013; Li et al., 2015). Besides, the previous studies were all observational studies with a limited sample size, which is susceptible to confounding and reverse causality. Thus, whether circulating adiponectin levels have causal effect on risk of developing RA remains unclear.

Mendelian randomization (MR) utilizes genetic variants as instrumental variables (IVs), which is more dependable to test the latent causal association between exposures and diseases (Smith and Ebrahim, 2003; Lawlor et al., 2008; Burgess et al., 2015; Bennett and Holmes, 2017). MR analysis can overcome the reverse causality as the genotypes are essentially fixed since conception, and they are prior to the disease occurrence process (Lawlor et al., 2008; Pierce and Burgess, 2013). Moreover, MR analyses can eliminate the confounder on account of random assignment of alleles. In the present study, we selected available IVs based on the currently published genome-wide association studies (GWASs) to explore whether circulating adiponectin levels were causally associated with RA using bidirectional two-sample MR analysis.

## Materials and Methods

### Study Design

**FIGURE 1:**
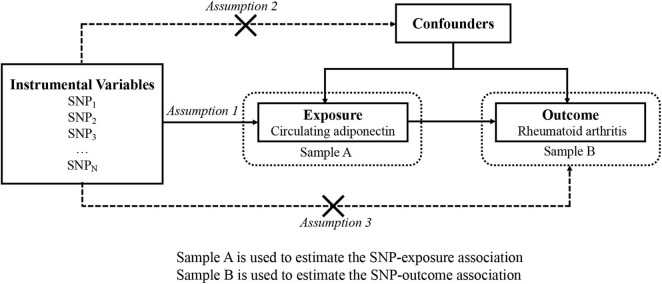
An overview of the study design with three fundamental assumptions about MR study.

The overview of this research design is illustrated in Figure 1. Implementation of the MR analysis depends on three stringent assumptions:

• Each genetic variant should be closely related to circulating adiponectin levels. We employed the *F* statistic and *R*^2^ to evaluate the strength of instruments and to evaluate the changes in circulating adiponectin expounded by the IV. If *F* statistic > 10 (Burgess and Thompson, 2011), it is considered that the association is strong enough to avoid weak instrument bias.
• Each genetic variant should not be associated with confounders of the exposure–outcome association.
• Each genetic variant should influence the risk for RA only by adiponectin instead of other pathway (no “pleiotropy”) (Bowden et al., 2016).

### Data Sources

Our investigation did not require further ethical approval because this MR research was implemented on the basis of publicly available data. We selected 20 single nucleotide polymorphisms (SNPs) associated with adiponectin as IVs from the latest GWAS meta-analysis, including 67,739 individuals of participants (89% European ancestry). These SNPs for adiponectin are genome-wide significant (*p* < 5 × 10^–8^) and not in linkage disequilibrium (LD) (Dastani et al., 2012). Relevant genetic information was gathered for the 20 selected SNPs, such as effect alleles, non-effect alleles, effect allele frequency, effect size (β), standard error, and *p*-value. Of note, the standard errors that are not directly provided in the original paper were estimated based on the β and *p*-values.

The RA data were extracted from a large independent meta-analysis of interracial GWAS with more than 100,000 participants with European and Asian ancestry (Okada et al., 2014). To decrease the risk of population stratification bias, merely summary-level data from European individuals (14,361 cases and 43,923 controls) were obtained for analysis (Okada et al., 2014). RA patients had been certified by rheumatoid arthritis specialists or met the diagnostic criteria for rheumatism (Arnett et al., 1988). Detailed information of above-mentioned GWASs in this study was accessible from previous publications (Okada et al., 2014).

To validate the results, we retrieved two additional data sources for RA, the FinnGen consortium (R5 release, 2021) and the UK Biobank (Sudlow et al., 2015). In the FinnGen consortium, we used the fifth wave of the GWAS results on RA, including 6,236 cases and 147,221 controls. All cases were defined by the code M13 in International Classification of Diseases---Tenth Revision. In the UK Biobank study, we leveraged GWAS data on 5,201 RA cases and 457,732 controls, which were deposited in the MRC-IEU.^1^ Due to the limited number of diagnosed RA cases available in the UK Biobank, we used self-reported RA data to increase the sample size.

### Linkage Disequilibrium Score Regression

To research the extent to which heritability of circulating adiponectin trait and RA is shared, we conducted LD score regression analysis via the LD Hub (Zheng et al., 2017). Compared with MR, LD score regression generally focuses on the number of genetic correlations, which could distinguish population stratification and polygenicity in GWAS.

### Bidirectional Mendelian Randomization

To assess whether reverse causality exists, MR was employed in taking RA as exposure and the circulating adiponectin level as the outcome. Since summary-level data used to extract the SNP for adiponectin were not available, another GWAS summary data on adiponectin from ADIPOGen consortium were used, which included 39,883 individuals (Dastani et al., 2012).

### Statistical Analyses

To sustain effect alleles always in connection with the same allele, we further adjusted the adiponectin and RA datasets. In the main analysis, the fixed-effects inverse-variance weighted (IVW) was performed to calculate the estimation of causal effect for different SNPs (Hartwig et al., 2016). Although numerous variants in an MR analysis can increase statistical power, pleiotropic genetic variation may lead to the presence of ineffective IVs (Hartwig et al., 2016). To adjust the potential pleiotropy, sensitivity analyses were utilized. More specifically, the WM method is effective in generating a consistent causal estimate when nearly one-half of the genetic variants were invalid IVs (Bowden et al., 2016). In the MR-Egger regression analysis (Bowden et al., 2015), we tested the existence of directional pluripotent effects on the basis of its intercept term (*p* < 0.05). The slope coefficient from Egger regression can be interpreted as the consistency of a causal estimate (Burgess and Thompson, 2017). Of note, such method may produce wider CIs with relatively lower precision (Bae and Lee, 2019b). To further test the existence of potential pleiotropy effects, we employed the MR-Pleiotropy residual sum and Outlier (MR-PRESSO) for outlier correction (Verbanck et al., 2018). In addition, hundreds of SNPs as IVs can be evaluated in the analysis by using the contamination mix method, which is not affected by the presence of invalid SNPs (Burgess et al., 2020).

Moreover, we manually searched secondary phenotypes of each selected SNP and its proxy in the PhenoScanner^2^ to further rule out potential pleiotropic effects (Supplementary Tables 1, 2). All the above MR analyses were reiterated after exclusion of SNPs associated with potential confounders at the genome-wide significance level.

To verify the statistical heterogeneity among SNPs used in IVW, Cochran’s Q test was employed (Egger et al., 1997). To probe the potential effect of an SNP on the causal estimates, we performed a ‘‘leave-one-out’’ sensitivity analysis with each SNP removed. The *a priori* statistical power was estimated using an online tool.^3^ Finally, the 20 SNPs for the adiponectin explained 1.17% of the variance. For a genetically predicted 1-SD increase in the circulating adiponectin level, our analyses had adequate power (>80%) to detect an OR of 1.29 for RA.

All statistical analyses were conducted in R (version 3.6.3, using the “TwoSampleMR,” “MendelianRandomization,” and “MRPRESSO” R packages). Statistical significance was set as bilateral *p*-values < 0.05.

## Results

### Linkage Disequilibrium Score Regression

Our analyses showed no significant genetic correlation Between circulating adiponectin levels and RA (*rG* = 0.127, 95% CI: –0.012 to 0.266, *P* = 0.074).

### Adiponectin and Rheumatoid Arthritis

A total of 20 independent SNPs were used as IVs in MR analysis. Summary information about these 20 SNPs is shown in Supplementary Tables 3–5. The *F* statistics for these SNPs are >10, demonstrating that the weak IV bias did not exist.

**FIGURE 2:**
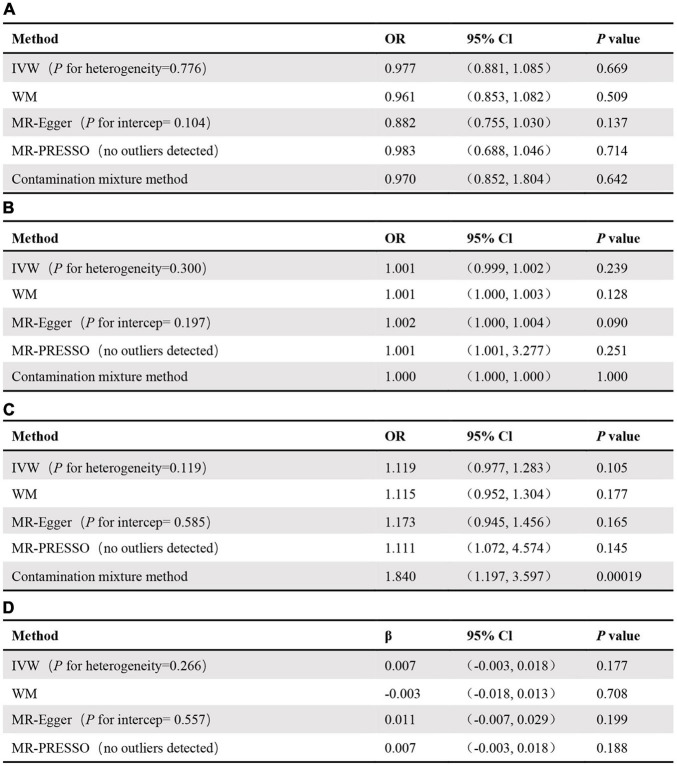
MR results for association of adiponectin and original-RA **(A)**, the UK Biobank-RA **(B)**, the FinnGen consortium-RA **(C)**, as well as association of RA and adiponectin **(D)**.

No significant relationship was observed between circulating adiponectin and the risk of RA [odds ratio (OR): 0.98, 95% confidence interval (CI): 0.88–1.09, *p* = 0.669] using the IVW method. Moreover, MR-Egger (OR: 0.88, 95% CI: 0.75–1.03, *p* = 0.137), weighted median (OR: 0.96, 95% CI: 0.85–1.08, *p* = 0.509), MR-PRESSO (OR: 0.98, 95% CI: 0.69–1.05, *p* = 0.713), and the contamination mix method (OR: 0.97, 95% CI: 0.85–1.80, *p* = 0.642) demonstrated consistent results (Figure 2A). The “leave-one-out analysis” indicated that no SNP can significantly promote the estimates of adiponectin on risk of RA, which showed that the results were reliable. We additionally discovered that several SNPs were related to obesity-related traits, such as high-density lipoprotein, low-density lipoprotein, and body mass index. Additionally, there was no evidence supporting a significant intercept about horizontal pleiotropy based on MR-Egger regression (intercept = 0.014, *p* = 0.104). No significant heterogeneity was found in the included independent SNP estimates using Cochran’s Q test. We applied the same analysis to the other two databases, which showed consistent results. The results of the analysis of the UK Biobank data and the FinnGen consortium are shown in Figures 2B,C, respectively.

### Rheumatoid Arthritis and Adiponectin

Similarly, we took RA as the exposure and circulating adiponectin levels as the outcome to verify whether RA was causally associated with circulating adiponectin levels. In the end, 29 SNPs with significant association (*p* < 5 × 10^–8^) for RA were included in this MR analysis (Supplementary Table 6). Based on the results, we observed no significant causal link between risk of RA and circulating adiponectin levels (β: 0.007, 95% CI: –0.003 to 0.018, *p* = 0.177) using the IVW method (Supplementary Figure 1), MR-Egger regression (β: 0.011, 95% CI: –0.007 to 0.029, *p* = 0.199), weighted-median method (β:–0.003, 95% CI: –0.018 to 0.013, *p* = 0.708), and MR PRESSO (β: 0.007, 95% CI: –0.003 to 0.018, *p* = 0.188) (Figure 2D). The leave-one-out analysis also confirmed that no SNP can drive the estimates of risk of RA on adiponectin (Supplementary Figure 2). In addition, there was no indication of a significant intercept about horizontal pleiotropy (intercept = –0.001, *p* = 0.557) (Supplementary Figure 3) and significant heterogeneity (Supplementary Figure 4).

## Discussion

The result of the current research shows no indication supporting the fact that circulating adiponectin levels were causally associated with RA risk. Besides, the genetic susceptibility of RA was not causally associated with circulating adiponectin levels.

Adiponectin is a secretion of adipose tissue and plays an important role in regulating the inflammatory response in inflammation (Tsatsanis et al., 2005; Fantuzzi, 2008). Some observational studies showed that serum adiponectin levels were significantly higher in RA patients compared with healthy controls (Alkady et al., 2011; Khajoei et al., 2019). In contrary, a case-control study documented that plasma adiponectin levels were significantly lower in RA patients compared with healthy controls in Chinese population (Zhao et al., 2020). A previous meta-analysis has revealed that adiponectin levels in the RA group were significantly higher than those in the control group, and adiponectin may have an antiphlogistic effect in the pathogenesis of RA by merging 11 studies (Lee and Bae, 2018). Although accumulated evidence suggested that elevated serum adiponectin levels are associated with the risk of RA, the causal influence between the two remains not known. There was no evidence supporting the fact that circulating adiponectin levels were causally associated with RA by using MR analysis in our study. The discrepancy between the results of our study and those of previous studies may be due to the bias or confounders intrinsic to observational epidemiological studies, such as a small sample size, heterogeneity in demographic characteristics, reverse causation, and selection bias.

As far as we know, it is considered to be the first MR study to explore the bidirectional relationship between circulating adiponectin levels and the risk of RA. There are some limitations to be aware of. First, satisfying three assumptions is a prerequisite for obtaining effective results in MR studies. Nevertheless, due to the flaws of MR analysis, the second hypothesis and the third hypothesis cannot be accurately accessed (Arnett et al., 1988), which may lead to potential deviations. The second hypothesis states that genetic variants that should influence the risk of RA only depend on risk factors instead of any other pathway. Thus, we used ME-Egger to identify horizontal pleiotropy. In addition, the third hypothesis states that each genetic variant should not be associated with confounders of the risk factor–outcome association, and we manually excluded SNPs associated with confounders to ensure that we obtained robust main results. Second, our research data were based upon four massive GWASs. Because the detailed demographic data and clinical symptoms of the subjects were not available, subgroup analysis cannot be performed. In addition, this study selected 20 limited SNPs related to circulating adiponectin levels as IVs, which only explain limit proportion of variation and may not have enough ability to find an association, so we sought two additional data sources, the FinnGen consortium and the UK Biobank, to validate the results. Finally, we could not explain the complex feedback loop because of the body’s adaptability to early physiological changes, which may add more potential interference to the IV analysis.

Although LD score regression analysis showed no statistically significant association between adiponectin and RA, it suggested a potential association as the *p*-value reached borderline significance. In previous studies, we found that adiponectin could be inhibited by inflammatory cytokines such as tumor necrosis factor-a and IL-6 in CD4^+^ T cells as well as 3T3-L1 adipocytes (Fasshauer et al., 2003; Tilg and Moschen, 2006). In addition, the inhibition of adiponectin may be mediated in part by the P44/42 MAP kinase (Fasshauer et al., 2003), which may result in lower adiponectin levels in RA patients than in healthy individuals. Some studies have found that adiponectin also stimulates the production of inflammatory cell factors such as IL-6, IL-8, and IL-11 in RA synovium fibroblasts (Liu et al., 2015), suggesting that adiponectin exacerbates inflammation. To verify the causal association between adiponectin and RA, MR studies with more powerful instruments and larger sample sizes are needed.

In summary, the above bidirectional MR study shows that genetically predicted circulating adiponectin levels are not causally associated with RA and do not support the causal relationship between RA and circulating adiponectin. Further studies are still demanded to authenticate our results.

## Data Availability Statement

The datasets presented in this study can be found in online repositories. The names of the repository/repositories and accession number(s) can be found in the article/Supplementary Material.

## Author Contributions

CF has came up with the idea and contributed to the design of this study. HC has conducted the main analysis and drafted the manuscript. All authors have revised the manuscript and approved the final version.

## Conflict of Interest

The authors declare that the research was conducted in the absence of any commercial or financial relationships that could be construed as a potential conflict of interest.

## Publisher’s Note

All claims expressed in this article are solely those of the authors and do not necessarily represent those of their affiliated organizations, or those of the publisher, the editors and the reviewers. Any product that may be evaluated in this article, or claim that may be made by its manufacturer, is not guaranteed or endorsed by the publisher.

## Footnotes

1
https://gwas.mrcieu.ac.uk/

2
http://www.phenoscanner.medschl.cam.ac.uk

3
https://sb452.shinyapps.io/power/
