# Supplementary material for: No Causal Association Between Adiponectin and the Risk of Rheumatoid Arthritis: A Mendelian Randomization Study
Source: Front Genet. 2021 Sep 24;12:670282. doi: 10.3389/fgene.2021.670282 (PMC8498100; doi:10.3389/fgene.2021.670282)

**Supplementary Materials**

Table S1: Potential secondary phenotypes of the genetic variants used for adiponectin

Table S2: Potential secondary phenotypes of the genetic variants used for RA

Table S3: (original-RA) Summary information on the SNPs used as genetic instruments for the adiponectin

Table S4: (the UK Biobank-RA) Summary information on the SNPs used as genetic instruments for the adiponectin

Table S5: (the FinnGen consortium-RA) Summary information on the SNPs used as genetic instruments for the adiponectin

Table S6: Summary information on the SNPs used as genetic instruments for the RA

Fig. S1: Forest plots of individual and summary estimates for causal associations between RA risk and adiponectin

Fig. S2: Leave-one-out plot to assess if a single SNP drives association between RA with adiponectin

Fig. S3: Scatter plots of causal associations of RA on adiponectin.

Fig. S4: Funnel plots of the causal association of RA and adiponectin to detect heterogeneity

Table S1: Potential secondary phenotypes of the genetic variants used for adiponectin

| SNP | Trait^1^ | *P* value |
| --- | --- | --- |
| Adiponectin |  |  |
| rs10282707 | High density lipoprotein | 1.03E-11 |
|  | Trunk fat-free mass | 3.51E-10 |
|  | Whole body water mass | 2.13E-08 |
|  | Arm predicted mass left | 3.44E-08 |
|  | Height | 2.97E-07 |
|  | Basal metabolic rate | 5.01E-07 |
|  | Impedance of arm left | 2.45E-06 |
|  | Total cholesterol | 3.99E-06 |
|  | Granulocyte count | 9.32E-06 |
| rs10861661 | Height | 8.10E-12 |
|  | Triglycerides | 2.60E-07 |
|  | High density lipoprotein | 5.05E-07 |
|  |  |  |
| rs11057353 | Waist hip ratio adjusted for BMI | 1.50E-06 |
|  | Body fat percentage | 2.61E-06 |
|  | Triglycerides | 4.32E-06 |
|  | Nonobstructive azoospermia | 6.14E-06 |
| rs11057405 | Hip circumference | 2.34E-30 |
|  | Trunk fat mass | 9.69E-24 |
|  | Weight | 8.05E-23 |
|  | Body fat percentage | 5.37E-18 |
|  | Basal metabolic rate | 7.35E-16 |
|  | Body mass index | 2.28E-14 |
|  | Whole body water mass | 5.91E-13 |
|  | High density lipoprotein | 1.23E-09 |
|  | Height | 3.15E-08 |
|  | Mean platelet volume | 1.33E-06 |
|  | Impedance of leg left | 2.48E-06 |
|  | Reticulocyte count | 9.31E-06 |
| rs11993554 | Platelet count | 4.01E-07 |
|  | Mean corpuscular volume | 2.69E-06 |
|  | Mean corpuscular hemoglobin | 3.75E-06 |
| rs13107325 | Extreme obesity with early age of onset | 1.50E-213 |
|  | Arm fat percentage right | 2.37E-30 |
|  | Hip circumference | 1.45E-20 |
|  | Hand grip strength right | 3.07E-20 |
|  | Usual walking pace | 3.92E-20 |
|  | Intelligence multi trait analysis | 3.00E-18 |
|  | Blood pressure | 2.00E-17 |
|  | Height | 5.37E-17 |
|  | Gene expression | 1.36E-16 |
|  | Impedance of leg right | 7.82E-16 |
|  | HDL cholesterol | 1.00E-15 |
|  | Self-reported osteoarthritis | 7.79E-15 |
|  | Hematocrit | 5.64E-14 |
|  | Alcohol intake frequency | 7.88E-14 |
|  | Body mass index | 1.50E-13 |
|  | Hemoglobin concentration | 5.83E-13 |
|  | Schizophrenia | 1.54E-12 |
|  | Platelet distribution width | 2.66E-12 |
|  | Weight | 3.24E-12 |
|  | Time spent watching television | 1.04E-10 |
|  | Sleep duration | 9.34E-10 |
|  | Types of physical activity in last 4 weeks: strenuous sports | 3.73E-09 |
|  | Pain type experienced in last month: none of the above | 1.05E-08 |
|  | Childhood BMI | 1.19E-08 |
|  | Qualifications: college or university degree | 1.19E-08 |
|  | Crohns disease | 2.40E-08 |
|  | Current employment status: in paid employment or self-employed | 6.39E-08 |
|  | Forced expiratory volume in 1-second, predicted | 7.40E-08 |
|  | Intelligence | 1.10E-07 |
|  | Taking other prescription medications | 1.54E-07 |
|  | Overall health rating | 2.48E-07 |
|  | Long-standing illness, disability or infirmity | 3.35E-07 |
|  | Self-reported urinary frequency or incontinence | 6.22E-07 |
|  | Types of transport used, excluding work: cycle | 6.83E-07 |
|  | Illnesses of siblings: none of the above, group 2 | 6.99E-07 |
|  | Sensitivity or hurt feelings | 1.18E-06 |
|  | Age started oral contraceptive pill | 1.20E-06 |
|  | Hair or balding pattern: pattern 4 | 1.31E-06 |
|  | Treatment with omega-3 or fish oil supplement | 1.67E-06 |
|  | Hayfever, allergic rhinitis or eczema | 1.95E-06 |
|  | Self-reported hiatus hernia | 5.14E-06 |
|  | Allergic disease | 6.65E-06 |
|  | Acquired deformities of fingers and toes | 7.35E-06 |
|  | log eGFR creatinine in non diabetics | 9.00E-06 |
| rs13133548 | Impedance of arm left | 5.51E-13 |
|  | Hip circumference | 3.16E-12 |
|  | High density lipoprotein | 5.21E-12 |
|  | Body fat percentage | 1.34E-11 |
|  | Triglycerides | 2.11E-07 |
|  | Waist circumference adjusted for smoking in females | 5.90E-06 |
| rs13303 | Reticulocyte count | 2.97E-14 |
|  | Impedance of whole body | 7.88E-13 |
|  | Body mass index | 4.28E-09 |
|  | Age at menarche | 7.49E-09 |
|  | Hemoglobin concentration | 5.58E-08 |
|  | Hematocrit | 6.88E-08 |
|  | Schizophrenia | 5.78E-07 |
|  | Cross disorder | 1.31E-06 |
|  | Systolic blood pressure | 1.89E-06 |
| rs145119400 | NA |  |
| rs17366568 | Blood protein levels | 6.00E-07 |
| rs2276853 | Lymphocyte count | 2.13E-16 |
|  | Platelet distribution width | 3.58E-13 |
|  | Monocyte percentage of white cells | 5.19E-06 |
|  | Heel bone mineral density | 6.73E-06 |
| rs2791552 | Hip circumference | 4.33E-29 |
|  | Impedance of arm left | 9.61E-25 |
|  | Whole body fat mass | 2.85E-13 |
|  | Diseases and traits | 5.00E-11 |
|  | log Fasting insulin | 7.63E-09 |
|  | Umbilical hernia | 8.20E-07 |
|  | Type II diabetes | 3.20E-06 |
|  | Falls in the last year | 3.70E-06 |
| rs2925979 | HDL cholesterol levels | 9.00E-22 |
|  | Impedance of arm right | 1.86E-12 |
|  | Vascular or heart problems diagnosed by doctor: none of the above | 2.08E-10 |
|  | Type II diabetes | 2.70E-08 |
| rs2943641 | Impedance of arm right | 9.89E-44 |
|  | Arm fat percentage left | 1.85E-15 |
|  | High density lipoprotein | 2.45E-15 |
|  | Trunk predicted mass | 2.26E-10 |
|  | Type II diabetes | 2.60E-09 |
|  | Hypertension | 3.14E-09 |
|  | Triglycerides | 9.91E-08 |
|  | Diastolic blood pressure | 1.57E-07 |
|  | Vascular or heart problems diagnosed by doctor: none of the above | 1.21E-06 |
|  | Whole body water mass | 1.22E-06 |
|  | Body mass index | 4.35E-06 |
|  | Reticulocyte count | 1.62E-06 |
|  | Number of treatments or medications taken | 9.53E-06 |
| rs3087866 | Height | 1.66E-15 |
|  | Pulse rate | 1.69E-15 |
|  | Reticulocyte count | 1.32E-11 |
|  | Impedance of arm left | 2.65E-11 |
|  | Primary sclerosing cholangitis | 1.09E-07 |
|  | Time spent using computer | 2.20E-07 |
|  | Frequency of tiredness or lethargy in last 2 weeks | 7.79E-07 |
| rs3735080 | Lymphocyte count | 3.25E-09 |
|  | Impedance of leg left | 3.86E-08 |
|  | Diastolic blood pressure | 1.70E-07 |
|  | Neutrophil percentage of granulocytes | 5.91E-06 |
| rs3865188 | Body mass index | 4.14E-07 |
|  | Whole body fat mass | 4.59E-07 |
|  | Hip circumference | 3.95E-06 |
|  | Weight | 9.66E-07 |
| rs4311394 | Reticulocyte count | 4.17E-09 |
|  | High density lipoprotein | 4.97E-09 |
|  | Leg fat percentage right | 2.03E-07 |
|  | log eGFR creatinine | 2.30E-07 |
|  | Waist circumference | 5.03E-07 |
|  | Type II diabetes | 1.20E-06 |
|  | Triglycerides | 2.07E-06 |
|  | Coronary heart disease | 8.50E-06 |
| rs4805885 | Impedance of arm right | 2.97E-16 |
|  | Body fat percentage | 7.81E-13 |
|  | High density lipoprotein | 7.25E-07 |
|  | Red blood cell count | 1.56E-06 |
|  | Comparative body size at age 10 | 4.39E-06 |
| rs7134375 | Cholesterol hdl | 8.38E-09 |
|  | Body mass index | 4.30E-07 |

Abbreviation: SNP, single nucleotide polymorphism; P-value: P-value for the genetic association

^1^ Similar traits were only listed once.

| Table S2: Potential secondary phenotypes of the genetic variants used for RA | | |
| --- | --- | --- |
| SNP | Trait ^1^ | *P* value |
| RA |  |  |
| rs10790268 | Allergic disease | 3.35E-10 |
|  | Self-reported asthma | 3.08E-08 |
| rs10985070 | Hayfever, allergic rhinitis or eczema | 3.12E-11 |
|  | Lymphocyte count | 3.88E-10 |
| rs11574914 | NA |  |
| rs12232497 | Sum basophil neutrophil counts | 1.06E-78 |
|  | No blood clot, bronchitis, emphysema, asthma, rhinitis, eczema or allergy diagnosed by doctor | 5.52E-23 |
|  | Inflammatory bowel disease | 1.02E-17 |
|  | Allergic disease | 7.08E-15 |
|  | Treatment with ventolin 100micrograms inhaler | 5.31E-13 |
|  | Primary biliary cirrhosis | 6.57E-12 |
|  | Ulcerative colitis | 1.87E-11 |
|  | High density lipoprotein | 2.80E-08 |
| rs12539741 | Systemic lupus erythematosus | 6.00E-31 |
| rs12764378 | Self-reported hypothyroidism or myxoedema | 9.44E-17 |
| rs13330176 | Monocyte count | 2.05E-11 |
| rs13426947 | Self-reported hypothyroidism or myxoedema | 5.73E-18 |
| rs1571878 | Self-reported hypothyroidism or myxoedema | 1.25E-11 |
| rs17264332 | NA |  |
| rs212389 | Sum neutrophil eosinophil counts | 1.32E-09 |
| rs225433 | NA |  |
| rs2561477 | Self-reported hypothyroidism or myxoedema | 3.29E-09 |
| rs2661798 | Monocyte count | 1.66E-09 |
| rs28411352 | NA |  |
| rs2844456 | Self-reported hypothyroidism or myxoedema | 7.24E-42 |
|  | White blood cell count | 1.23E-26 |
|  | Self-reported malabsorption or coeliac disease | 5.78E-24 |
|  | Treatment with methotrexate | 3.49E-21 |
|  | Asthma | 1.32E-19 |
|  | No blood clot, bronchitis, emphysema, asthma, rhinitis, eczema or allergy diagnosed by doctor | 3.18E-13 |
|  | Taking other prescription medications | 1.60E-11 |
|  | Peak expiratory flow | 1.88E-11 |
|  | Treatment with folic acid product | 3.27E-09 |
| rs3087243 | Graves disease | 1.00E-21 |
|  | Type 1 diabetes | 2.30E-17 |
|  | Hypothyroidism | 1.00E-15 |
|  | Selective immunoglobulin A deficiency IgAD | 1.20E-15 |
|  | Alopecia areata | 6.00E-12 |
| rs34046593 | Self-reported hypothyroidism or myxoedema | 3.64E-10 |
| rs34695944 | NA |  |
| rs4239702 | NA |  |
| rs4452313 | NA |  |
| rs56339890 | Reticulocyte count | 2.75E-10 |
|  | Hemoglobin concentration | 1.72E-08 |
| rs592390 | NA |  |
| rs60733400 | NA |  |
| rs62395855 | Age-related macular degeneration | 7.89E-33 |
|  | Body fat percentage | 1.30E-08 |
| rs6679677 | Self-reported hypothyroidism or myxoedema | 1.07E-122 |
|  | Selective immunoglobulin A deficiency IgAD | 6.00E-42 |
|  | Type 1 diabetes | 5.00E-26 |
|  | White blood cell count | 5.72E-21 |
|  | Treatment with methotrexate | 1.25E-16 |
|  | Systemic lupus erythematosus | 1.00E-15 |
|  | Crohns disease | 5.00E-09 |
| rs6936656 | Self-reported malabsorption or coeliac disease | 7.28E-17 |
|  | Started insulin within one year diagnosis of diabetes | 2.51E-12 |
|  | Self-reported hyperthyroidism or thyrotoxicosis | 6.68E-11 |
|  | Age-related macular degeneration | 1.32E-09 |
|  | Primary sclerosing cholangitis | 4.61E-09 |
|  | Treatment with dovonex scalp solution | 9.68E-09 |
|  | Height | 1.59E-08 |
|  | Psoriasis | 2.39E-08 |
| rs706778 | Self-reported hypothyroidism or myxoedema | 3.48E-13 |
|  | Primary sclerosing cholangitis | 5.48E-13 |
|  | Alopecia areata | 4.90E-10 |
|  | Pediatric autoimmune diseases | 6.00E-09 |
| rs8026898 | Hair or balding pattern: pattern 4 | 6.13E-11 |
|  | Height | 1.39E-08 |
| rs8032939 | Self-reported hypothyroidism or myxoedema | 2.23E-11 |
|  | Eosinophil percentage of white cells | 4.95E-08 |
| rs9275183 | Primary sclerosing cholangitis | 3.04E-35 |
|  | Diabetes diagnosed by doctor | 7.28E-32 |
|  | Eye problems or disorders: diabetes related eye disease | 1.05E-29 |
|  | Self-reported polymyalgia rheumatica | 7.07E-26 |
|  | Treatment with methotrexate | 5.21E-25 |
|  | IgA deficiency | 1.60E-18 |
|  | Nasal polyp | 8.34E-17 |
|  | Long-standing illness, disability or infirmity | 3.97E-16 |
|  | Asthma | 4.08E-16 |
|  | Treatment with folic acid product | 4.50E-12 |
|  | Number of treatments or medications taken | 2.45E-11 |
|  | Treatment with prednisolone | 2.63E-11 |
|  | Ulcerative colitis | 3.23E-11 |
|  | Eosinophil count | 3.78E-11 |
|  | Height | 7.53E-10 |
|  | Body mass index | 1.09E-09 |
| rs9603608 | Sum eosinophil basophil counts | 2.16E-10 |
|  | Eosinophil percentage of granulocytes | 1.23E-09 |
|  | Age at menarche | 3.39E-09 |
| rs9653442 | Intelligence multi trait analysis | 7.00E-16 |
|  | Years of educational attainment | 2.79E-14 |

Abbreviation: SNP, single nucleotide polymorphism; P-value: P-value for the genetic association

^1^ Similar traits were only listed once.

| Table S3: (original-RA) Summary information on the SNPs used as genetic instruments for the adiponectin | | | | | | | | | | |
| --- | --- | --- | --- | --- | --- | --- | --- | --- | --- | --- |
| SNP | EAF | Adiponectin | | | RA | |  | | *R^2^* ^1^ | *F*-statistic ^2^ |
|  |  | Beta | SE | *P* value | Beta | SE | | *P* value |  |  |
| Adiponectin |  |  |  |  |  |  | |  |  |  |
| rs10282707 | 0.38 | -0.040 | 0.006 | 1.96E-10 | -0.020 | 0.021 | | 0.340 | 0.0006 | 41 |
| rs10861661 | 0.23 | -0.040 | 0.007 | 1.75E-09 | 0.010 | 0.027 | | 0.710 | 0.0005 | 36 |
| rs11057353 | 0.65 | 0.054 | 0.006 | 2.74E-19 | -0.010 | 0.016 | | 0.530 | 0.0012 | 81 |
| rs11057405 | 0.09 | -0.077 | 0.010 | 2.90E-14 | -0.030 | 0.038 | | 0.420 | 0.0009 | 58 |
| rs11993554 | 0.10 | 0.030 | 0.010 | 3.75E-03 | 0.010 | 0.046 | | 0.830 | 0.0001 | 8 |
| rs13107325 | 0.05 | 0.072 | 0.014 | 1.05E-07 | -0.030 | 0.043 | | 0.480 | 0.0004 | 28 |
| rs13133548 | 0.49 | -0.039 | 0.006 | 5.91E-12 | -0.020 | 0.016 | | 0.210 | 0.0007 | 47 |
| rs2276853 | 0.59 | -0.033 | 0.006 | 3.35E-08 | 0.020 | 0.026 | | 0.450 | 0.0004 | 30 |
| rs2791552 | 0.68 | -0.052 | 0.007 | 1.75E-14 | -0.010 | 0.026 | | 0.700 | 0.0009 | 59 |
| rs2943641 | 0.65 | -0.058 | 0.006 | 1.76E-21 | -0.020 | 0.018 | | 0.260 | 0.0013 | 91 |
| rs3087866 | 0.79 | -0.040 | 0.007 | 2.14E-08 | -0.010 | 0.013 | | 0.440 | 0.0005 | 31 |
| rs3735080 | 0.25 | -0.049 | 0.007 | 7.70E-13 | 0.010 | 0.031 | | 0.750 | 0.0008 | 51 |
| rs3865188 | 0.47 | -0.034 | 0.006 | 1.14E-08 | -0.020 | 0.018 | | 0.280 | 0.0005 | 33 |
| rs4311394 | 0.26 | 0.258 | 0.040 | 1.68E-10 | -0.020 | 0.016 | | 0.210 | 0.0006 | 41 |
| rs4805885 | 0.60 | 0.063 | 0.006 | 2.85E-25 | 0.010 | 0.012 | | 0.410 | 0.0016 | 108 |
| rs10282707 | 0.38 | -0.040 | 0.006 | 1.96E-10 | -0.020 | 0.021 | | 0.340 | 0.0006 | 41 |
| Abbreviation: SNP, single nucleotide polymorphism; EAF, effect allele frequency; SE, standard error; RA, rheumatoid arthritis;  ^1^ *R^2^* were calculated using the following formula: (2×EAF×(1-EAF) × beta^2^)/[(2×EAF×(1-EAF) × beta^2^) + (2×EAF×(1-EAF) × N×SE^2^)], where EAF is the effect allele frequency, beta is the estimated effect on adiponectin, Ν is the sample size of the GWAS for the SNP-adipokine association and SE is the standard error of the estimated effect.  ^2^ *F* statistic were calculated using the following formula: *R^2^*(N-2)/(1-*R^2^*), where *R^2^* is the proportion of variance in adiponectin explained by each instrument and N is the sample size of the GWAS for the SNP-adipokine association. | | | | | | | | | | |

| Table S4: ((the UK Biobank-RA) Summary information on the SNPs used as genetic instruments for the adiponectin | | | | | | | | | | |
| --- | --- | --- | --- | --- | --- | --- | --- | --- | --- | --- |
| SNP | EAF | Adiponectin | | | RA | |  | | *R^2^* ^1^ | *F*-statistic ^2^ |
|  |  | Beta | SE | *P* value | Beta | SE | | *P* value |  |  |
| Adiponectin |  |  |  |  |  |  | |  |  |  |
| rs10282707 | 0.383 | -0.040 | 0.006 | 1.96E-10 | 0.032 | 0.021 | | 0.128 | 0.00009 | 41 |
| rs10861661 | 0.229 | -0.040 | 0.007 | 1.75E-09 | 0.023 | 0.026 | | 0.374 | 0.00008 | 36 |
| rs11057353 | 0.645 | 0.054 | 0.006 | 2.74E-19 | -0.011 | 0.021 | | 0.607 | 0.00017 | 81 |
| rs11057405 | 0.086 | -0.077 | 0.010 | 2.90E-14 | -0.035 | 0.034 | | 0.293 | 0.00012 | 58 |
| rs11993554 | 0.101 | 0.030 | 0.010 | 3.75E-03 | 0.010 | 0.037 | | 0.779 | 0.00002 | 8 |
| rs13107325 | 0.047 | 0.072 | 0.014 | 1.05E-07 | -0.001 | 0.086 | | 0.989 | 0.00006 | 28 |
| rs13133548 | 0.489 | -0.039 | 0.006 | 5.91E-12 | 0.036 | 0.020 | | 0.066 | 0.00010 | 47 |
| rs13303 | 0.569 | 0.058 | 0.006 | 2.69E-21 | 0.038 | 0.020 | | 0.051 | 0.00019 | 90 |
| rs145119400 | 0.005 | -0.300 | 0.042 | 8.95E-13 | -0.122 | 0.107 | | 0.255 | 0.00142 | 660 |
| rs17366568 | 0.118 | -0.226 | 0.009 | 1.43E-145 | -0.046 | 0.028 | | 0.107 | 0.00007 | 30 |
| rs2276853 | 0.594 | -0.033 | 0.006 | 3.35E-08 | 0.022 | 0.020 | | 0.282 | 0.00013 | 59 |
| rs2791552 | 0.676 | -0.052 | 0.007 | 1.75E-14 | -0.065 | 0.021 | | 0.002 | 0.00035 | 164 |
| rs2925979 | 0.693 | 0.080 | 0.006 | 1.38E-37 | 0.012 | 0.021 | | 0.565 | 0.00020 | 91 |
| rs2943641 | 0.646 | -0.058 | 0.006 | 1.76E-21 | -0.023 | 0.020 | | 0.248 | 0.00007 | 31 |
| rs3087866 | 0.786 | -0.040 | 0.007 | 2.14E-08 | -0.008 | 0.024 | | 0.722 | 0.00011 | 51 |
| rs3735080 | 0.245 | -0.049 | 0.007 | 7.70E-13 | 0.009 | 0.021 | | 0.671 | 0.00007 | 33 |
| rs3865188 | 0.472 | -0.034 | 0.006 | 1.14E-08 | 0.021 | 0.020 | | 0.291 | 0.00009 | 41 |
| rs4311394 | 0.257 | 0.258 | 0.040 | 1.68E-10 | 0.011 | 0.023 | | 0.627 | 0.00023 | 108 |
| rs4805885 | 0.603 | 0.063 | 0.006 | 2.85E-25 | -0.001 | 0.020 | | 0.942 | 0.00018 | 85 |
| rs7134375 | 0.410 | 0.053 | 0.006 | 3.69E-20 | 0.009 | 0.020 | | 0.644 | 0.00009 | 41 |
| Abbreviation: SNP, single nucleotide polymorphism; EAF, effect allele frequency; SE, standard error; RA, rheumatoid arthritis;  ^1^ *R^2^* were calculated using the following formula: (2×EAF×(1-EAF)×beta^2^)/[(2×EAF×(1-EAF)×beta^2^) + (2×EAF×(1-EAF)×N×SE^2^)], where EAF is the effect allele frequency, beta is the estimated effect on adiponectin, Ν is the sample size of the GWAS for the SNP-adipokine association and SE is the standard error of the estimated effect.  ^2^ *F* statistic were calculated using the following formula: *R^2^*(N-2)/(1-*R^2^*), where *R^2^* is the proportion of variance in adiponectin explained by each instrument and N is the sample size of the GWAS for the SNP-adipokine association. | | | | | | | | | | |

| Table S5: (the FinnGen consortium-RA) Summary information on the SNPs used as genetic instruments for the adiponectin | | | | | | | | | | |
| --- | --- | --- | --- | --- | --- | --- | --- | --- | --- | --- |
| SNP | EAF | Adiponectin | | | RA | |  | | R2 1 | F-statistic 2 |
|  |  | Beta | SE | P value | Beta | SE | | P value |  |  |
| Adiponectin |  |  |  |  |  |  | |  |  |  |
| rs10282707 | 0.383 | -0.040 | 0.006 | 1.96E-10 | 0.032 | 0.021 | | 0.128 | 0.0003 | 41 |
| rs10861661 | 0.229 | -0.040 | 0.007 | 1.75E-09 | 0.023 | 0.026 | | 0.374 | 0.0002 | 36 |
| rs11057353 | 0.645 | 0.054 | 0.006 | 2.74E-19 | -0.011 | 0.021 | | 0.607 | 0.0005 | 81 |
| rs11057405 | 0.086 | -0.077 | 0.010 | 2.90E-14 | -0.035 | 0.034 | | 0.293 | 0.0004 | 58 |
| rs11993554 | 0.101 | 0.030 | 0.010 | 3.75E-03 | 0.010 | 0.037 | | 0.779 | 0.0001 | 8 |
| rs13107325 | 0.047 | 0.072 | 0.014 | 1.05E-07 | -0.001 | 0.086 | | 0.989 | 0.0002 | 28 |
| rs13133548 | 0.489 | -0.039 | 0.006 | 5.91E-12 | 0.036 | 0.020 | | 0.066 | 0.0003 | 47 |
| rs13303 | 0.569 | 0.058 | 0.006 | 2.69E-21 | 0.038 | 0.020 | | 0.051 | 0.0006 | 90 |
| rs145119400 | 0.005 | -0.300 | 0.042 | 8.95E-13 | -0.122 | 0.107 | | 0.255 | 0.0003 | 51 |
| rs17366568 | 0.118 | -0.226 | 0.009 | 1.43E-145 | -0.046 | 0.028 | | 0.107 | 0.0043 | 660 |
| rs2276853 | 0.594 | -0.033 | 0.006 | 3.35E-08 | 0.022 | 0.020 | | 0.282 | 0.0002 | 30 |
| rs2791552 | 0.676 | -0.052 | 0.007 | 1.75E-14 | -0.065 | 0.021 | | 0.002 | 0.0004 | 59 |
| rs2925979 | 0.693 | 0.080 | 0.006 | 1.38E-37 | 0.012 | 0.021 | | 0.565 | 0.0011 | 164 |
| rs2943641 | 0.646 | -0.058 | 0.006 | 1.76E-21 | -0.023 | 0.020 | | 0.248 | 0.0006 | 91 |
| rs3087866 | 0.786 | -0.040 | 0.007 | 2.14E-08 | -0.008 | 0.024 | | 0.722 | 0.0002 | 31 |
| rs3735080 | 0.245 | -0.049 | 0.007 | 7.70E-13 | 0.009 | 0.021 | | 0.671 | 0.0003 | 51 |
| rs3865188 | 0.472 | -0.034 | 0.006 | 1.14E-08 | 0.021 | 0.020 | | 0.291 | 0.0002 | 33 |
| rs4311394 | 0.257 | 0.258 | 0.040 | 1.68E-10 | 0.011 | 0.023 | | 0.627 | 0.0003 | 41 |
| rs4805885 | 0.603 | 0.063 | 0.006 | 2.85E-25 | -0.001 | 0.020 | | 0.942 | 0.0007 | 108 |
| rs7134375 | 0.410 | 0.053 | 0.006 | 3.69E-20 | 0.009 | 0.020 | | 0.644 | 0.0006 | 85 |
| Abbreviation: SNP, single nucleotide polymorphism; EAF, effect allele frequency; SE, standard error; RA, rheumatoid arthritis;  1 R2 were calculated using the following formula: (2×EAF×(1-EAF)×beta2)/[(2×EAF×(1-EAF)×beta2) + (2×EAF×(1-EAF)×N×SE2)], where EAF is the effect allele frequency, beta is the estimated effect on adiponectin, Ν is the sample size of the GWAS for the SNP-adipokine association and SE is the standard error of the estimated effect.  2 F statistic were calculated using the following formula: R2(N-2)/(1-R2), where R2 is the proportion of variance in adiponectin explained by each instrument and N is the sample size of the GWAS for the SNP-adipokine association. | | | | | | | | | | |

| Table S6: Summary information on the SNPs used as genetic instruments for the RA | | | | | | | | | |
| --- | --- | --- | --- | --- | --- | --- | --- | --- | --- |
| SNP | EAF | RA | | | Adiponectin | | | *R^2^* ^1^ | *F*-statistic ^2^ |
|  |  | Beta | SE | *P* value | Beta | SE | *P* value |  |  |
| RA |  |  |  |  |  |  |  |  |  |
| rs10790268 | 0.19 | 0.163 | 0.021 | 3.30E-15 | -0.001 | 0.006 | 0.81 | 0.0011 | 62 |
| rs10985070 | 0.53 | -0.083 | 0.015 | 1.70E-08 | 0.003 | 0.004 | 0.57 | 0.0005 | 32 |
| rs11574914 | 0.64 | 0.122 | 0.017 | 1.50E-13 | 0.001 | 0.005 | 0.89 | 0.0009 | 55 |
| rs12232497 | 0.53 | 0.094 | 0.016 | 3.60E-09 | 0.000 | 0.004 | 0.91 | 0.0006 | 35 |
| rs12539741 | 0.17 | 0.157 | 0.023 | 1.20E-11 | -0.002 | 0.007 | 0.81 | 0.0008 | 46 |
| rs12764378 | 0.78 | 0.131 | 0.018 | 1.90E-13 | 0.009 | 0.006 | 0.12 | 0.0009 | 54 |
| rs13330176 | 0.79 | 0.113 | 0.020 | 9.00E-09 | 0.007 | 0.007 | 0.29 | 0.0006 | 33 |
| rs13426947 | 0.23 | 0.131 | 0.019 | 2.40E-12 | -0.014 | 0.006 | 0.02 | 0.0008 | 49 |
| rs1571878 | 0.41 | -0.117 | 0.015 | 4.90E-15 | -0.005 | 0.004 | 0.23 | 0.0011 | 61 |
| rs17264332 | 0.17 | 0.163 | 0.018 | 7.10E-19 | -0.000 | 0.005 | 0.93 | 0.0013 | 79 |
| rs212389 | 0.55 | 0.095 | 0.016 | 1.10E-09 | 0.006 | 0.005 | 0.26 | 0.0006 | 37 |
| rs225433 | 0.19 | -0.128 | 0.023 | 1.80E-08 | -0.001 | 0.009 | 0.90 | 0.0005 | 32 |
| rs2561477 | 0.62 | -0.105 | 0.017 | 5.20E-10 | -0.006 | 0.005 | 0.19 | 0.0007 | 39 |
| rs2661798 | 0.38 | 0.094 | 0.015 | 1.10E-09 | 0.003 | 0.004 | 0.55 | 0.0006 | 37 |
| rs28411352 | 0.71 | 0.104 | 0.018 | 5.20E-09 | 0.006 | 0.005 | 0.27 | 0.0006 | 34 |
| rs2844456 | 0.05 | 0.892 | 0.029 | 1.00E-200 | 0.055 | 0.071 | 0.45 | 0.0158 | 933 |
| rs3087243 | 0.54 | -0.139 | 0.015 | 9.20E-20 | 0.003 | 0.004 | 0.52 | 0.0014 | 83 |
| rs34046593 | 0.37 | 0.140 | 0.017 | 9.20E-17 | -0.001 | 0.005 | 0.85 | 0.0012 | 69 |
| rs34695944 | 0.60 | 0.117 | 0.015 | 4.40E-14 | 0.003 | 0.005 | 0.57 | 0.0010 | 57 |
| rs4239702 | 0.74 | 0.139 | 0.018 | 4.20E-14 | 0.003 | 0.006 | 0.58 | 0.0010 | 57 |
| rs4452313 | 0.33 | 0.105 | 0.017 | 2.70E-10 | -0.002 | 0.005 | 0.75 | 0.0007 | 40 |
| rs56339890 | 0.98 | -0.400 | 0.065 | 7.10E-10 | -0.036 | 0.016 | 0.03 | 0.0007 | 38 |
| rs592390 | 0.57 | -0.095 | 0.016 | 3.80E-09 | 0.007 | 0.004 | 0.14 | 0.0006 | 35 |
| rs60733400 | 0.32 | -0.105 | 0.017 | 1.30E-09 | 0.001 | 0.005 | 0.88 | 0.0006 | 37 |
| rs62395855 | 0.98 | -0.616 | 0.065 | 4.40E-21 | -0.024 | 0.021 | 0.26 | 0.0015 | 89 |
| rs6679677 | 0.86 | 0.593 | 0.023 | 3.10E-149 | 0.019 | 0.007 | 0.01 | 0.0115 | 677 |
| rs6936656 | 0.06 | -0.446 | 0.041 | 5.10E-27 | 0.000 | 0.011 | 0.97 | 0.0020 | 116 |
| rs706778 | 0.45 | 0.104 | 0.015 | 7.10E-12 | -0.005 | 0.004 | 0.27 | 0.0008 | 47 |
| rs8026898 | 0.75 | 0.148 | 0.018 | 2.40E-17 | -0.000 | 0.005 | 1.00 | 0.0012 | 72 |
| rs8032939 | 0.65 | 0.117 | 0.017 | 2.40E-12 | 0.004 | 0.005 | 0.41 | 0.0008 | 49 |
| rs9275183 | 0.18 | 0.777 | 0.023 | 1.00E-200 | -0.002 | 0.007 | 0.72 | 0.0192 | 1144 |
| rs9603608 | 0.45 | -0.104 | 0.016 | 7.60E-11 | -0.000 | 0.005 | 0.95 | 0.0007 | 42 |
| rs9653442 | 0.43 | -0.105 | 0.015 | 3.60E-12 | 0.002 | 0.004 | 0.73 | 0.0008 | 48 |
| Abbreviation: SNP, single nucleotide polymorphism; EAF, effect allele frequency; SE, standard error; RA, rheumatoid arthritis;  ^1^ *R^2^* were calculated using the following formula: (2×EAF×(1-EAF)×beta^2^)/[(2×EAF×(1-EAF)×beta^2^) + (2×EAF×(1-EAF)×N×SE^2^)], where EAF is the effect allele frequency, beta is the estimated effect on adiponectin, Ν is the sample size of the GWAS for the SNP-adipokine association and SE is the standard error of the estimated effect.  ^2^ *F* statistic were calculated using the following formula: *R^2^*(N-2)/(1-*R^2^*), where *R^2^* is the proportion of variance in adiponectin explained by each instrument and N is the sample size of the GWAS for the SNP-adipokine association. | | | | | | | | | |
|  | | | | | | | | | |

Fig. S1: Forest plots of individual and summary estimates for causal associations between RA risk and adiponectin


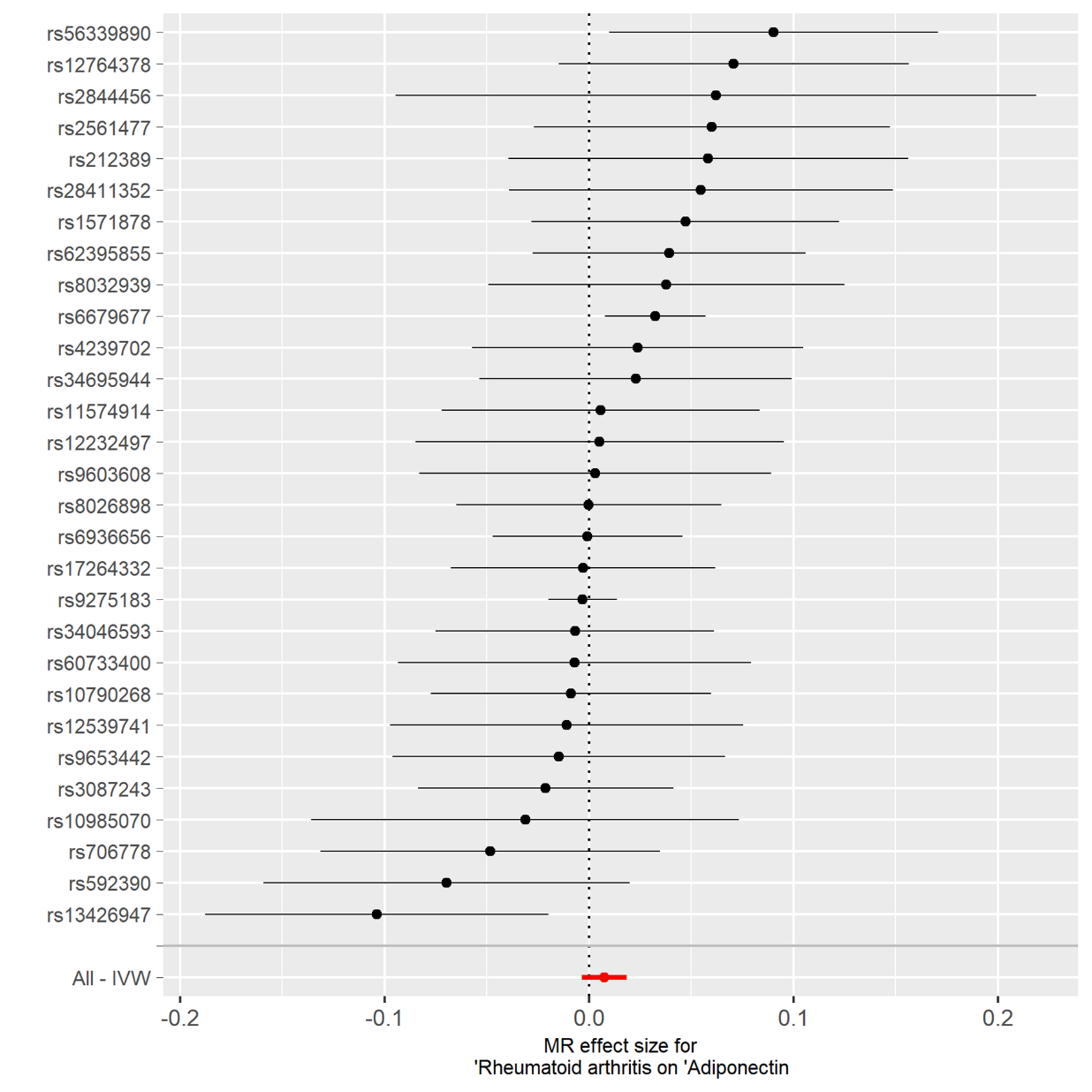


Fig. S2: Leave-one-out plot to assess if a single SNP drives association between RA with adiponectin


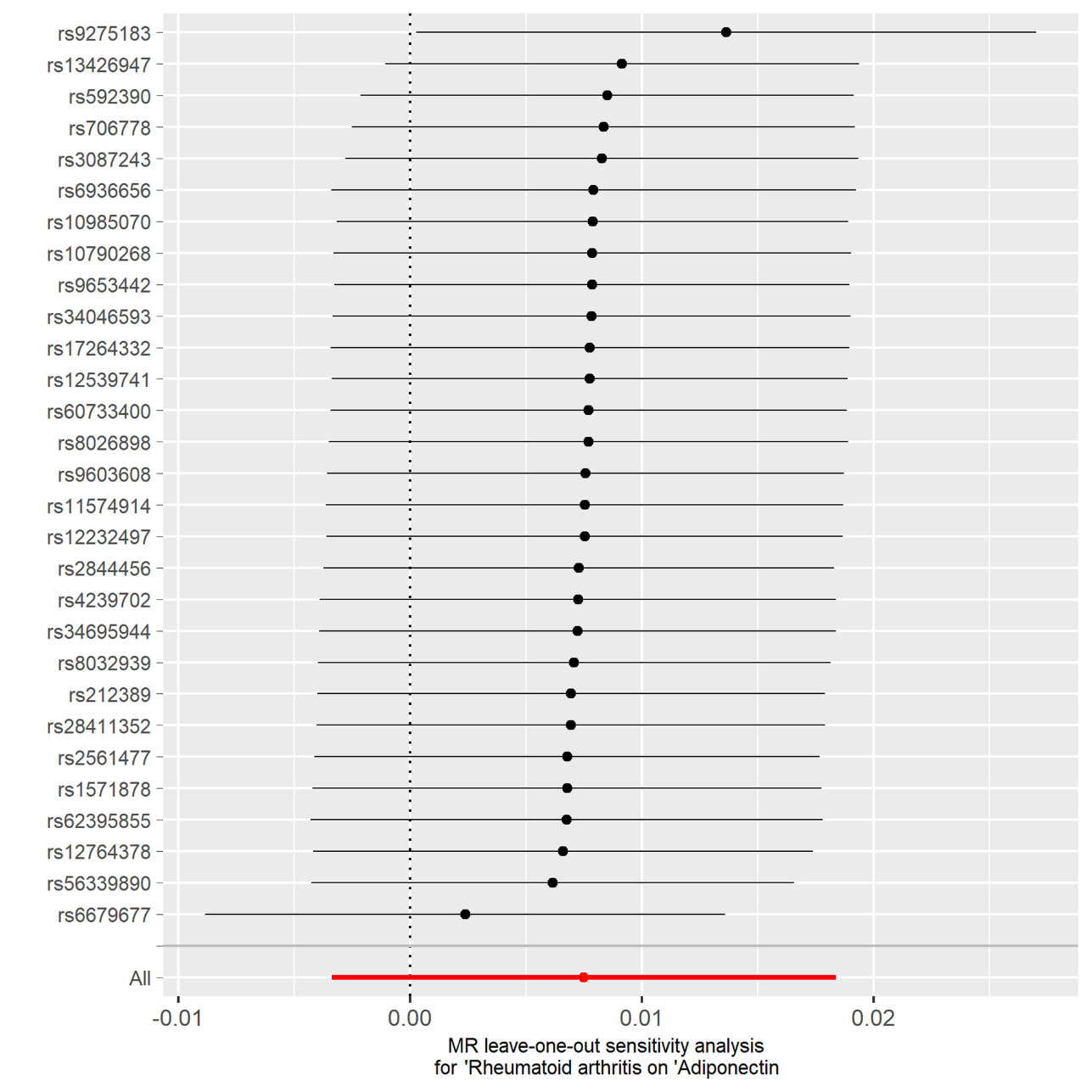


Fig. S3: Scatter plots of causal associations of RA on adiponectin.


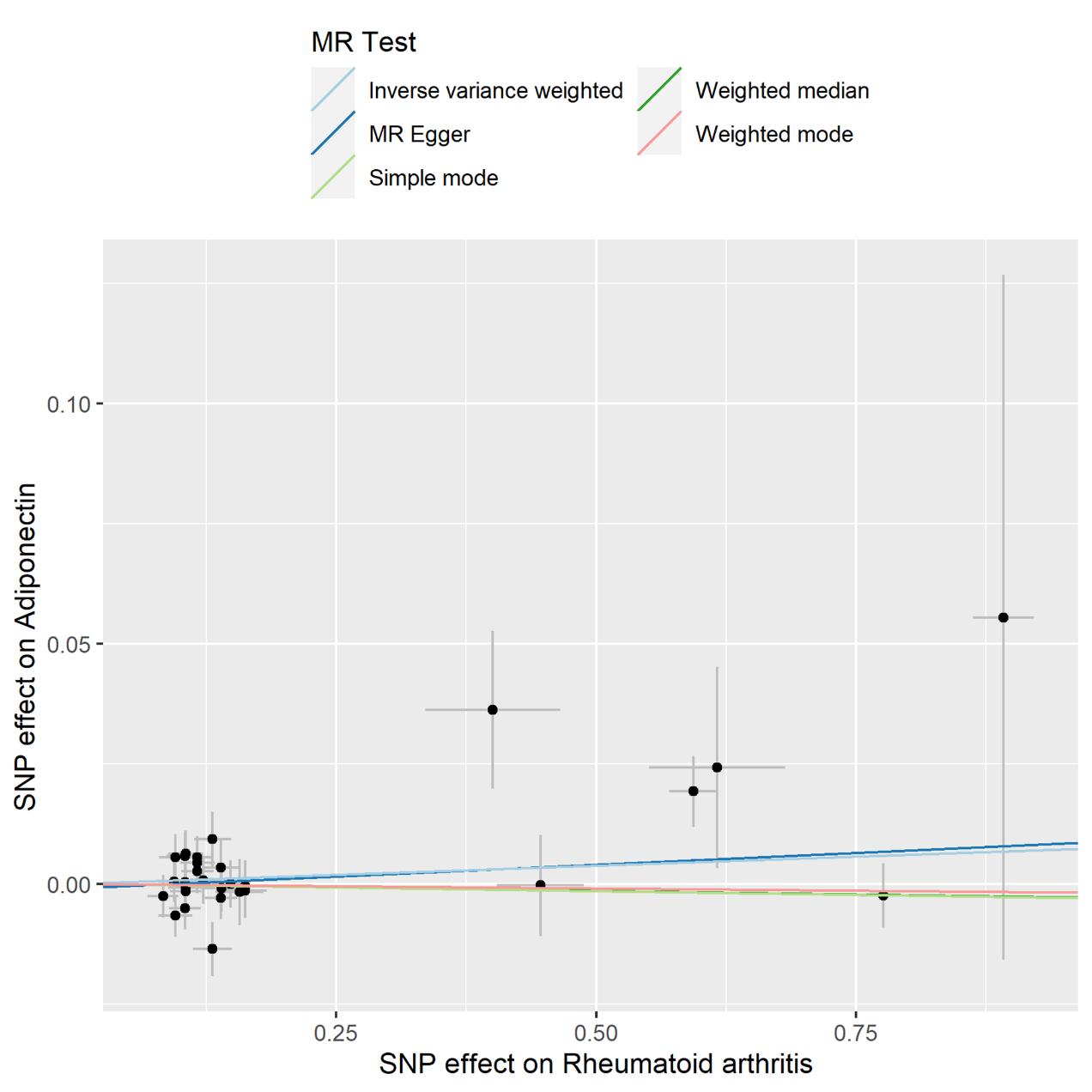


Fig. S4: Funnel plots of the causal association of RA and adiponectin to detect heterogeneity


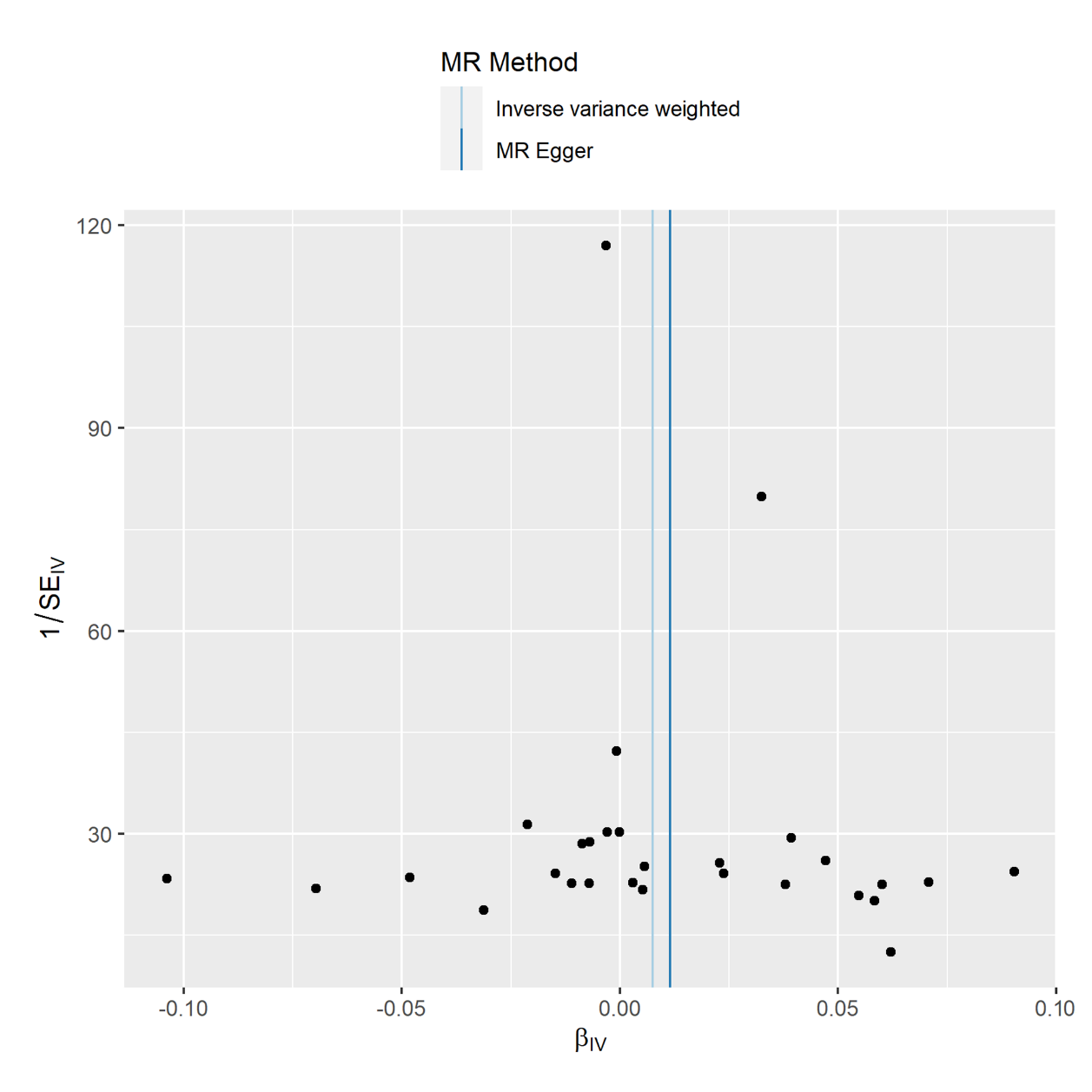

Supplement: Supplementary file 1 [file Data_Sheet_1.docx]
